# Analysis of the epidemic situation of respiratory pathogens in central and southern China before and after the COVID-19 pandemic

**DOI:** 10.3389/fped.2026.1826861

**Published:** 2026-05-15

**Authors:** Zhuoya Xiao, Linfei Yang, Ting Shi, Yuqi She, Zhimin Wan, Yufang Hu, Qiong Liu

**Affiliations:** 1 Clinical Laboratory of Hunan Provincial People’s Hospital, The First Affiliated Hospital of Hunan Normal University, Changsha, Hunan, China; 2 Department of Transfusion Medicine, Xiangya Hospital, Central South University, Hunan, China; 3 Faculty of Materials Science and Chemical Engineering, Ningbo University, Ningbo, Zhejiang, China

**Keywords:** children, COVID-19, epidemiological analysis, pediatric critical care, respiratory pathogens

## Abstract

**Background:**

Respiratory tract infection is a common disease leading to pediatric hospitalization, exhibiting different epidemiological trends with seasons and ages. This study retrospectively analyzed the infection status of 12 respiratory pathogens from 2018 to 2023, and examined the changes before and after the COVID-19 pandemic.

**Methods:**

A total of 51,597 hospitalized children with suspected respiratory tract infection, who underwent multiplex PCR testing in a tertiary hospital in Changsha from 2018 to 2023 were included. Testing items included respiratory syncytial virus, adenovirus, influenza virus, parainfluenza virus, Mycoplasma pneumoniae, Chlamydia pneumoniae, and bacterial culture results, among others.

**Results:**

The infant group had the highest positive rate at 39.25% (7,729/19,693), and the school-age group the lowest at 25.34% (2,015/7,951) (*P* < 0.05). During 2020–2021, pathogen positive rates declined compared with pre-pandemic levels; a peak of FluA, FluB, and PIV1-3 occurred in late 2022. After restriction lifting in 2023, FluA, FluB, PIV1, PIV3, AdV, and RSV all rebounded. Among bacterial pathogens, Streptococcus pneumoniae accounted for 26.35% (2,104/7,983), followed by Haemophilus influenzae (20.11%) and Moraxella catarrhalis (14.93%) (*P* < 0.005). Co-infection analysis revealed significant positive interactions among Escherichia coli, Klebsiella pneumoniae, and Acinetobacter baumannii, and between Pseudomonas aeruginosa and A. baumannii/K. pneumoniae (*φ*>0.9, *P* < 0.05). M. pneumoniae was more likely to co-infect with viral than bacterial pathogens.

**Conclusion:**

Due to the impact of the epidemic, the positive rate and disease spectrum of respiratory pathogens have changed. The pandemic induced an initial suppression followed by a marked resurgence of RSV, influenza, and M. pneumoniae after restrictions were lifted. Segmented regression confirmed RSV exhibited the steepest post-pandemic rebound, and toddlers born during lockdown showed a disproportionate surge in infections, supporting the “immunity debt” hypothesis. These findings underscore the importance of age-specific surveillance for preventing pediatric critical illness.

## 1 Introduction

Respiratory tract infections (RTIs) are common causes of hospitalization in children and can lead to severe outcomes, including life-threatening complications. Due to their weak immunity and frequent exposure to crowded, poorly ventilated environments, children are susceptible to large-scale outbreaks within a short period. Pathogens spread through direct or indirect contact with droplets or contaminated surfaces (1), resulting in a high incidence among children aged 0–3 years. Studies have shown that in 2019, respiratory infections ranked second among the leading causes of death in children aged 0–10 years, imposing a heavy economic burden on affected families (2).

Viruses have become a predominant cause of childhood pneumonia globally, particularly in low- and middle-income countries, as evidenced by major multi-country studies. It is estimated that the annual global incidence of viral community-acquired pneumonia reaches hundreds of millions of cases (3). Common viral pathogens include respiratory syncytial virus (RSV), adenovirus (AdV), influenza virus (Flu), and parainfluenza viruses (PIVs). Parainfluenza viruses are classified into four serotypes; however, type 4 (PIV4) is excluded from this study as it typically causes mild upper respiratory tract diseases (4). Thus, this research only includes the first three types: type 1 (PIV1), type 2 (PIV2), and type 3 (PIV3). In addition to viruses, atypical pathogens are also significant contributors to infections, with Mycoplasma pneumoniae (MP) and Chlamydia pneumoniae (Cpn) being the most common. The prevalence of these pathogens varies by region, season, and age (5). Following the COVID-19 pandemic, respiratory infections have attracted increasing public attention (6). Due to the abuse of antibiotics, the detection rate of bacterial co-infections in the upper respiratory tract has increased compared to previous periods. Moreover, a considerable proportion of infected children develop severe illness, such as pediatric acute respiratory distress syndrome (ARDS), which requires intensive care and prolonged mechanical ventilation, further increasing the risk of morbidity and mortality (7).

Recent studies have reported that from 2019 to 2021, the implementation of measures such as mask-wearing and mandatory lockdowns in China led to a decrease in the detection rate of common RTI pathogens (8). After the government announced the removal of “nucleic acid testing requirements” in late 2022, a large-scale COVID-19 outbreak occurred in China (9). With the relaxation of epidemic prevention measures, respiratory viruses may experience a more intense rebound (10), potentially leading to an increase in severe pediatric cases. There are few reports on the patterns of respiratory infections before and after the pandemic in Changsha, and pathogen infections are influenced by factors such as age, season, and geography. This study aims to analyze the epidemiological characteristics to clarify the dynamic changes in childhood RTIs before and after the COVID-19 pandemic, with a particular focus on severe infections, thereby providing references for the prevention of common respiratory pathogens, early identification of high-risk children, and public health decision-making in the future.

## 2 Materials and methods

Hospitalized pediatric patients with suspected respiratory tract infections who underwent multiplex PCR(Polymerase Chain Reaction) testing for respiratory pathogens in a tertiary hospital in Changsha from 2018 to 2023 were selected. Blood samples and respiratory specimens of the patients were collected for detection. According to the diagnostic codes of the International Classification of Diseases, 10th Revision, Clinical Modification (ICD-10-CM) Version 2.0, adenovirus (AdV) infection was coded as B34.000, respiratory syncytial virus (RSV) infection as B34.800 × 003, influenza A/B (FluA/B) infection as J10.0-J10.8, parainfluenza virus (PIV) infection as B34.800 × 002, Mycoplasma pneumoniae (MP) infection as A49.300 and J15.700, and Chlamydia pneumoniae (Cpn) infection as J16.0. Real-time fluorescent quantitative PCR (Polymerase Chain Reaction) was used to detect the positivity or negativity of pathogens in respiratory specimens. By combining PCR technology with real-time fluorescent quantitative technology, the amplification of viral nucleic acids was monitored in real-time to achieve quantitative analysis. The instrument used was QuantStudio 5 from ABI (USA) with its matching reagents. For immune indicators, the instrument used was iFlash3000-H automatic chemiluminescence immunoassay analyzer from Shenzhen YHLO Biotech Co., Ltd. with its matching reagents. The detection principle was that the solid-phase antigen formed an immune complex with the antibody to be tested; after a series of chemical reactions, the markers in the immune complex generated chemiluminescent signals, and the concentration of relevant indicators was determined by the intensity of the optical signals. Bacterial identification was performed using VITEK MS mass spectrometer from bioMérieux (France). It should be noted that the detection of Mycoplasma pneumoniae (MP) in this study included both direct pathogen nucleic acid detection via multiplex PCR and serological antibody testing (MP-IgM). These two assays were performed concurrently as independent diagnostic items throughout the entire study period (2018–2023). The nucleic acid test indicates the presence of the pathogen itself, whereas the IgM antibody test reflects the host's recent immune response.Data collection was completed using Excel. In this study, categorical variables such as gender, age, and admission year were presented as percentages. For non-ordinal categorical variables, chi-square test or Fisher's exact test was used for inter-group comparison; for ordinal categorical variables and non-normally distributed variables, non-parametric tests such as Wilcoxon test or Kruskal–Wallis test were used for comparison. Graphs were plotted using GraphPad Prism 10.0, and all data analyses were performed using SPSS 27.0.

## 3 Results

### 3.1 Positive rate of non-bacterial respiratory pathogens

**Figure 1:**
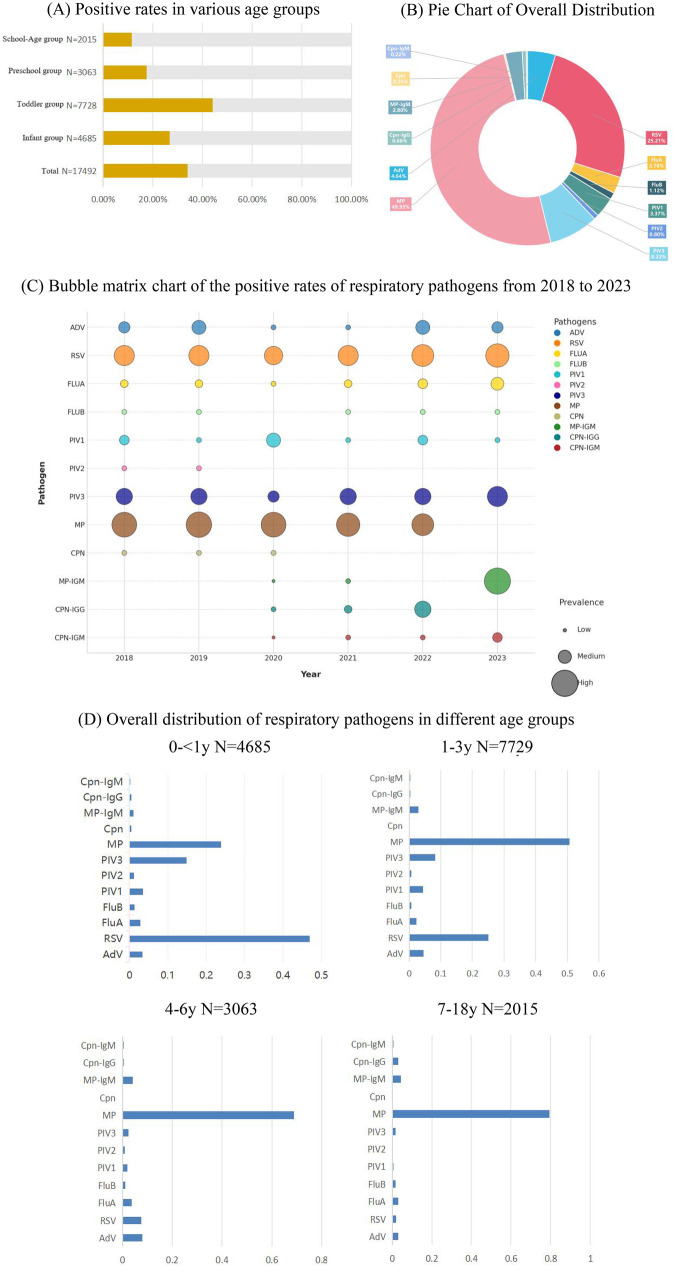
**(A)** Bar chart showing the total positive rates and positive cases in different age groups from 2018 to 2023.the toddler group (1–3 years) consistently exhibited the highest positive rate across all study years, peaking at 39.25%, highlighting this age bracket as the primary reservoir of respiratory pathogen circulation. **(B)** Pie chart showing the positive proportion of all pathogens from 2018 to 2023. **(C)** Bubble matrix chart of the positive rates of respiratory pathogens from 2018 to 2023, where bubbles of different colors represent different types of pathogens. In 2023, a substantial increase in MP-IgM seropositivity was observed, and thus it is highlighted in this panel to represent the M. pneumoniae epidemic trend for that year, while the nucleic acid detection rate for MP decreased markedly. Notably, both MP and MP-IgM tests were available throughout the study period. **(D)** Overall distribution of respiratory pathogens in different age.groups.Age-stratified pathogen profiles revealing distinct susceptibility patterns: RSV dominates in infants (0–<1 year), accounting for nearly half of all detected pathogens, whereas M. pneumoniae becomes the predominant pathogen in school-age children, reflecting age-dependent shifts in host immunity and exposure risk.

Over the six years from 2018 to 2023, a total of 51,597 cases were included in this study. The overall positive rate of all non-bacterial pathogens was 33.90% (17,492/51,597). The toddler group had the highest positive rate at 39.25% (7,729/19,693), followed by the infant group at 33.55% (4,685/13,966), the preschool children group at 30.67% (3,063/9,987), and the school-age children group with the lowest at 25.34% (2,015/7,951) (see Figure 1A). Among the 12 pathogens, Mycoplasma pneumoniae (MP) had the highest positive rate (49.93%), followed by respiratory syncytial virus (RSV) (25.21%), parainfluenza virus type 3 (PIV3) (8.22%), adenovirus (AdV) (4.64%), parainfluenza virus type 1 (PIV1) (3.37%), MP-IgM (2.80%), influenza A (FluA) (2.78%), influenza B (FluB) (1.12%), parainfluenza virus type 2 (PIV2) (0.80%), Chlamydia pneumoniae (Cpn)-IgG (0.66%), Cpn (0.25%), and Cpn-IgM (0.22%) (see Figure 1B).The incidence and changing trends of different pathogens over the six years are shown in Figure 1C. The detection rates of respiratory pathogens were generally higher before the pandemic (2018–2019). During the pandemic (2020–2022), the detection rate of MP showed little change in the first two years but decreased in 2022. The main pathogens before the pandemic were MP, RSV, PIV3, and AdV; during the pandemic, they were MP, RSV, PIV3, PIV1, and AdV; and after the pandemic, they were RSV, MP-IgM, PIV3, FluA, and AdV.The positive rate of PIV3 decreased in 2020 (3.05%) with little change in other years. The positive rate of RSV gradually increased after the outbreak of the pandemic (22.39% vs. 29.89% vs. 31.83% vs. 44.33%, *P* < 0.001). The positive rate of PIV1 increased in the early stage of the pandemic (11.51% vs. 1.01%, *P* < 0.001), and the positive rate of FluA increased after the pandemic (3.41% vs. 6.35%, *P* < 0.001). In 2022, the positive rates of influenza viruses and parainfluenza viruses basically returned to the pre-pandemic levels.Affected by the pandemic, the number of patients who underwent multiplex PCR testing for respiratory pathogens in 2020–2021 decreased compared with 2018–2019, while the overall positive rate increased. From 2022 to the end of the pandemic in 2023, the number of patients tested increased, and the overall positive rate decreased (see Table 1 for details). However, the positive rates of RSV, FluA, PIV3, MP-IgM, and Cpn-IgM in 2023 were higher than those in 2022, with statistically significant differences (*P* < 0.001).

**Table 1:** Distribution of total cases and positive rates from 2018 to 2023 (*N*, %).

Detection results	2018	2019	2020	2021	2022	2023	X^2^	*P*
Total	8955	13013	7488	7359	4733	10049		
N	3364 (37.57)	5238 (40.25)	3336 (44.55)	3339 (45.37)	1115 (25.56)	1040 (10.35)	3818.67	<0.001

The pathogens of patients in different age groups over the six years were statistically analyzed. Infants and young children were high-risk groups for infections, and the susceptible pathogens varied among different age groups (see Figure 1D and Table 2).

**Table 2:** Detection of non-bacterial pathogens in different age groups from 2018 to 2023.

Positive	Total 51597	0-<1y 13966	1-3y 19693	4-6y 9987	7-18y 7951	X^2^	*P*
+	17492	4685	7729	3063	2015		
AdV	811	161	346	244	60	103.547	**<0**.**001**
RSV	4410	2202	1935	231	42	2123.175	**<0**.**001**
FluA	487	135	177	116	59	9.029	**<0**.**001**
FluB	195	61	59	38	37	6.114	0.106
PIV1	590	168	343	65	14	150.02	**<0**.**001**
PIV2	140	53	55	29	3	22.252	**<0**.**001**
PIV3	1437	695	631	76	35	573.06	**<0**.**001**
MP	8734	1117	3909	2107	1601	1093.2	**<0**.**001**
Cpn	43	20	22	0	1	21.027	**<0**.**001**
MP-IgM	490	48	227	128	87	76.62	**<0**.**001**
Cpn-IgG	116	24	15	16	61	127.28	**<0**.**001**
Cpn-IgM	39	1	10	13	15	27.66	**<0**.**001**

Bold numbers indicate significant differences.

To further investigate the hypothesis that prolonged low exposure during the pandemic may have altered population susceptibility, we specifically examined the 1–3 year age group in 2023—a cohort born during the period of strictest lockdown measures (2020–2021) with presumably minimal prior pathogen exposure. When compared with the same age group in the pre-pandemic year 2019, the positive rate of RSV among toddlers in 2023 increased significantly (18.26% vs. 7.79%, *χ*^2^ = 120.38, *P* < 0.001), and the positive rate of PIV3 also demonstrated a substantial rise (6.52% vs. 3.27%, *χ*^2^ = 29.48, *P* < 0.01). In contrast, the school-age children group (7–18 years) in 2023 did not exhibit a comparable surge in RSV or PIV3 infections relative to 2019 levels (*P* > 0.05). This cohort-specific elevation in viral positivity among the youngest children in 2023 provides epidemiological evidence supporting a pandemic-associated immunity gap.

Except for FluB, the differences among other pathogens were statistically significant (*P* < 0.001). Young children account for 44.19% of the total number of positive patients (7729/17492). MP, RSV, and PIV3 are the three non-bacterial pathogens with the highest overall detection rates. Among them, MP can be detected in all age groups, and its positive rate increases with age. MP is the main pathogen causing respiratory infections in children aged 4–18 years. In infants aged 0-<1 year, various pathogens could be detected, with RSV having the highest detection rate, accounting for 49.93% (2202/4410). Except for MP, the positive rates of other respiratory pathogens gradually decreased with age.

### 3.2 Epidemic patterns of respiratory non-bacterial pathogens

**Figure 2:**
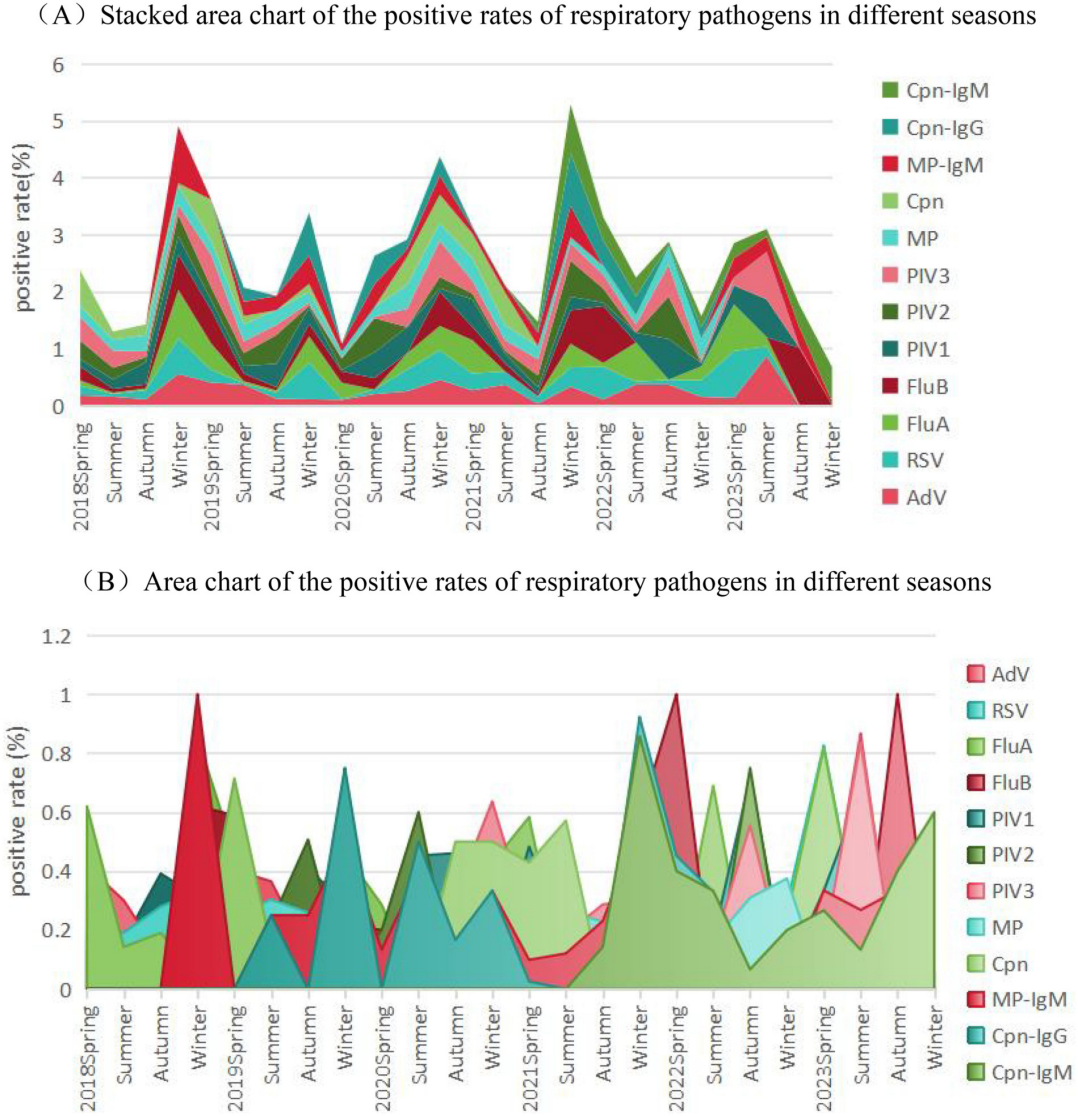
Seasonal trends in the positive rates of respiratory pathogens from 2018 to 2023. **(A)** Stacked area chart of the positive rates of 12 respiratory pathogens across different seasons. The total height of the stack at each season indicates the cumulative positive rate of all pathogens, while the height of each colored band corresponds to the positive rate of a single pathogen. **(B)** Area chart of the positive rates of individual respiratory pathogens across different seasons. Each colored area depicts the seasonal fluctuation in the positive rate of a specific pathogen. Overlapping areas may occur, but the vertical axis value always reflects the positive rate of each individual pathogen.

Seasons are divided in accordance with international law: spring (March-May), summer (June-August), autumn (September-November), and winter (December-February of the following year). This study excluded the results of January and February 2018 and analyzed the epidemic patterns of different pathogens from 2018 to 2023. The seasonal data of all positive cases are shown in Figures 2A,B. During the study period, it was observed that the incidence of most pathogens was the highest in autumn and winter, and the lowest in summer. Among them, AdV and MP had no obvious seasonal specificity, could prevail throughout the year, and MP had a relatively high positive rate in all four seasons. At the end of the 2022 epidemic, there was a peak in positive cases of FluA, FluB, and PIV1-3; after the lifting of restrictions in 2023, the positive rates of various pathogens such as FluA, FluB, PIV1, PIV3, AdV, and RSV showed an upward trend compared with those during the epidemic period.

Of note, MP was excluded from this segmented regression analysis because its nucleic acid detection positivity rate dropped precipitously to zero in 2023, whereas the seropositivity rate of MP-IgM surged abnormally to 28.75%. The epidemic intensities reflected by these two testing modalities are not directly comparable across the time series. To quantify the stage-specific impact of the COVID-19 pandemic on the epidemiological trajectories of respiratory pathogens, segmented linear regression models were fitted to the annual positive rates of five major respiratory viruses (RSV, PIV3, FluA, AdV, and PIV1). The models included a continuous time variable (0 for 2018, increasing by 1 annually), a pandemic phase dummy variable (0 for pre-pandemic 2018–2019, 1 for pandemic and post-pandemic 2020–2023), and their interaction term. For RSV, the pre-pandemic trend was significantly negative (*β* = –5.33% per year, *P* = 0.049), whereas the interaction term indicated a significant steepening of the post-pandemic slope (*β* =  + 7.20% per year, *P* = 0.041), resulting in a net upward trend of +1.87% per year after 2020. PIV3 showed a stable pre-pandemic trend (*β* = –1.48% per year, *P* = 0.520) with a positive but non-significant post-pandemic slope increase (*β* =  + 3.97% per year, *P* = 0.233). Influenza A exhibited a marginally increasing pre-pandemic trend (*β* =  + 1.74% per year, *P* = 0.100), a marked immediate drop at pandemic onset (*β* = –3.17%, *P* = 0.076), and no significant change in the subsequent slope (*P* = 0.726 for interaction). Adenovirus, which had been rising prior to the pandemic (*β* =  + 4.36% per year, *P* = 0.062), experienced a sharp level decline during the pandemic period (*β* = –8.18%, *P* = 0.044) without a significant slope alteration thereafter (*P* = 0.532). PIV1 displayed an isolated surge in 2020 (immediate level change *β* =  + 9.19%, *P* = 0.130) but the interaction term was not significant (*P* = 0.666), suggesting a transient outbreak. These quantitative estimates confirm that the pandemic and associated control measures exerted differential perturbations on the transmission trajectories of respiratory viruses, with RSV demonstrating the most pronounced post-pandemic rebound in slope. Detailed regression parameters are provided in Table 3.

**Table 3:** Parameters of segmented linear regression analysis for five major respiratory viruses.

Pathogen	Parameter	Coefficient (*β*)	*P*-value
RSV	Intercept (β₀)	0.2509	**0**.**002**
	Time (β₁)	−0.0533	**0**.**049**
	Period (β₂)	0.0766	0.057
	Interaction (β₃)	0.0720	**0**.**041**
PIV3	Intercept (β₀)	0.0942	**0**.**039**
	Time (β₁)	−0.0148	0.520
	Period (β₂)	−0.0488	0.241
	Interaction (β₃)	0.0397	0.233
FluA	Intercept (β₀)	0.0217	0.068
	Time (β₁)	0.0174	0.100
	Period (β₂)	−0.0317	0.076
	Interaction (β₃)	0.0029	0.726
AdV	Intercept (β₀)	0.0490	0.050
	Time (β₁)	0.0436	0.062
	Period (β₂)	−0.0818	**0**.**044**
	Interaction (β₃)	0.0105	0.532
PIV1	Intercept (β₀)	0.0232	0.429
	Time (β₁)	−0.0131	0.635
	Period (β₂)	0.0919	0.130
	Interaction (β₃)	−0.0146	0.666

Note:Model: Y = β₀ + β₁×Time + β₂×Period + β₃×(Time × Period) + *ε*.

Time: coded as 0 for 2018, +1 for each subsequent year.

Bold font indicates *P* < 0.05.

Period: 0 for 2018–2019, 1 for 2020–2023.

Coefficients are expressed as decimals (e.g., 0.2509 corresponds to 25.09%).

### 3.3 Characteristics of bacterial infections

**Figure 3:**
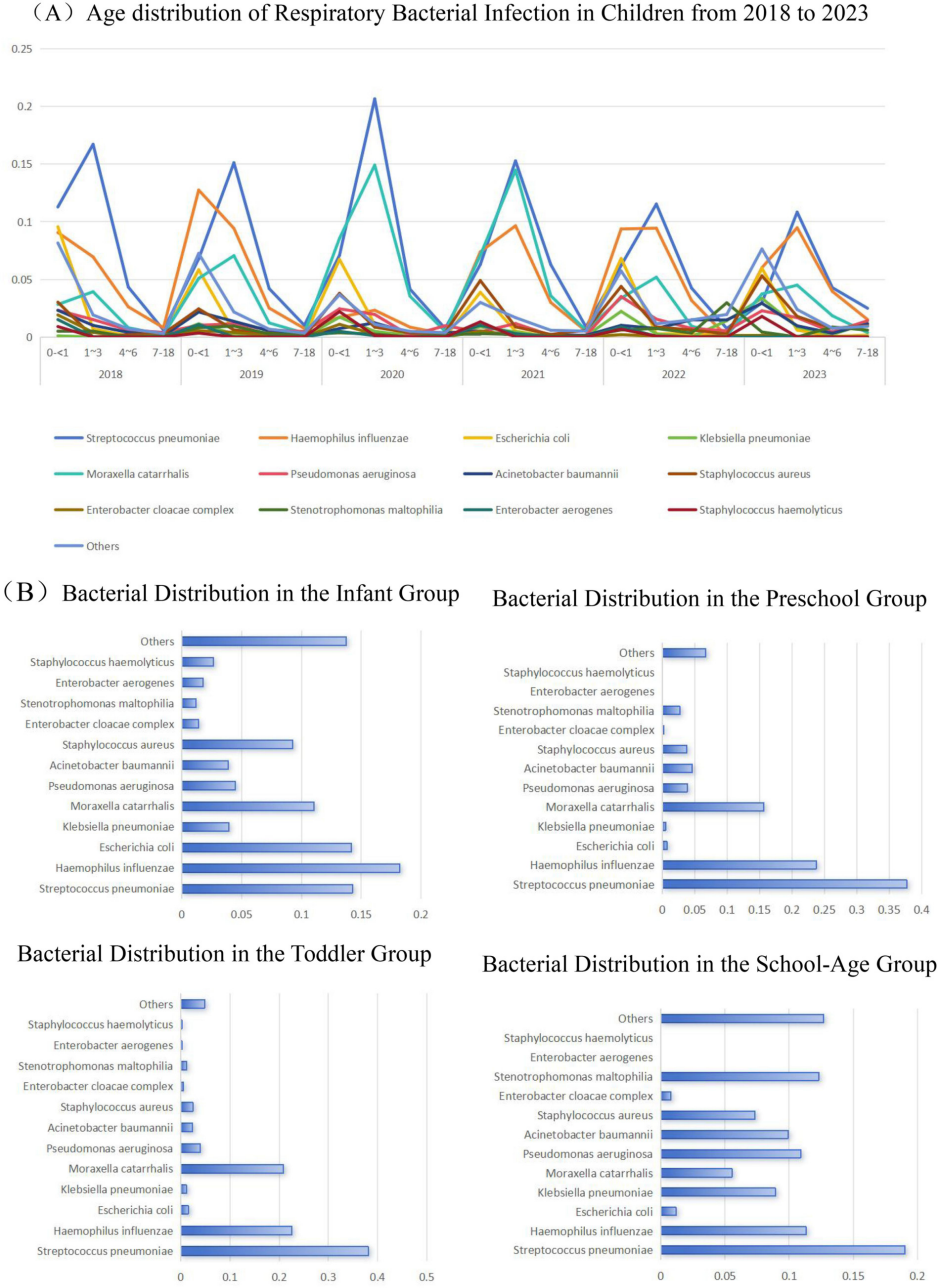
**(A)** line graph of age distribution of respiratory bacterial infections from 2018 to 2023. Age-specific bacterial detection rates showing that S. pneumoniae and H. influenzae are the leading pathogens in children under 6 years, whereas opportunistic Gram-negative bacteria such as P. aeruginosa and A. baumannii become increasingly prevalent in school-age children, signaling a shift in microbial ecology with advancing age.**(B)** Bar graph of the overall distribution of respiratory bacteria in different age groups.

A total of 7,983 patients with concurrent bacterial infections were identified in this study. Among them, the detection rate was the highest in the infant group, accounting for 44.17% (3,526/7,983), followed by the toddler group at 37.33% (2,980/7,983), the preschool—age children group at 12.19% (973/7,983), and the school—age children group had the lowest rate at 6.31% (504/7,983). According to the overall distribution, the main pathogenic bacteria with the highest detection rates were *Streptococcus pneumoniae*(S. pneumoniae), *Haemophilus influenzae*(H. influenzae), *Escherichia coli*(E. coli), *Klebsiella pneumoniae*(K. pneumoniae), etc. The overall distribution of these bacteria is shown in Table 4 and Figure 3A below.

**Table 4:** Age distribution of bacterial pathogens.

Bcteria	0-<1y	1-3y	4–6y	7–18y	X^2^/Z	*P*
*S. pneumoniae*	503	1138	367	96	325.272	**<0**.**001**
*H. influenzae*	643	674	231	57	281.951	**<0**.**001**
*E. coli*	500	45	7	6	1119.719	**<0**.**001**
*K.pneumoniae*	139	34	5	45	170.922	**<0**.**001**
*M. catarrhalis*	389	623	152	28	240.080	**<0**.**001**
*P. aeruginosa*	158	118	37	55	54.710	**<0**.**001**
*A. baumannii*	137	71	45	50	57.850	**<0**.**001**
*S. aureus*	327	74	36	37	426.486	**<0**.**001**
Enterobacter cloacae complex	49	16	2	4	65.226	**<0**.**001**
*S. maltophilia*	41	35	26	62	67.052	**<0**.**001**
*E. aerogenes*	62	6	0	0	142.421	**<0**.**001**
*S.haemolyticus*	93	2	0	0	241.908	**<0**.**001**
*Others*	485	144	65	64	531.832	**<0**.**001**

Bold numbers indicate significant differences.

In respiratory tract specimens, *S. pneumoniae* had the highest detection rate of bacterial infections, accounting for 26.35% (2,104/7,983), followed by *H. influenzae* at 20.11% (1,605/7,983) and *Moraxella catarrhalis(M. catarrhalis)* at 14.93% (1,192/7,983), with statistically significant differences (*P* < 0.005). Further age—specific analysis showed that bacterial infections were mainly distributed in the 0–3 - year - old infant and toddler group, and the infection rate in the group of children over 3 years old decreased with increasing age. Among them, *S. pneumoniae, H. influenzae,* and *M. catarrhalis* mainly occurred in toddlers aged 1–3 years, while other bacteria were more common in infants aged 0 to < 1 year; *Enterobacter aerogenes(E. aerogenes)* and *Staphylococcus haemolyticus(S. haemolyticus)* were found in the infant group. The top 5 bacteria causing respiratory tract infections in the infant group were *H. influenzae* accounting for 18.24% (643/3,526), *S. pneumoniae* at 14.27% (503/3,526), *E.coli* at 14.18% (500/3,526), *M. catarrhalis* at 11.03% (389/3,526), and *Staphylococcus aureus(S. aureus)* at 9.27% (327/3,526). The top three pathogenic bacteria in the toddler group and the preschool—age children group were the same, namely *S. pneumoniae*, *H. influenzae*, and *M. catarrhalis*. The top five pathogenic bacteria in the school—age children group changed, which were *S. pneumoniae* accounting for 19.05% (96/504), *Stenotrophomonas maltophilia(S. maltophilia)* at 12.30% (62/504), *H. influenzae* at 11.31% (57/504), *P. aeruginosa* at 10.91% (55/504), and *Acinetobacter baumannii(A. baumannii)* at 9.92% (50/504), as detailed in Figure 3B below. The positive rate of *S. pneumoniae* remained high over the six years. During the early stage of the 2020–2021 epidemic, the positive rate of *M. catarrhalis* increased in toddlers aged 1–3 years, and it continued to decrease from the late stage of the epidemic until its end (14.47% VS 5.19% VS 4.50%, *P* < 0.001).

### 3.4 Characteristics of coinfection

**Figure 4:**
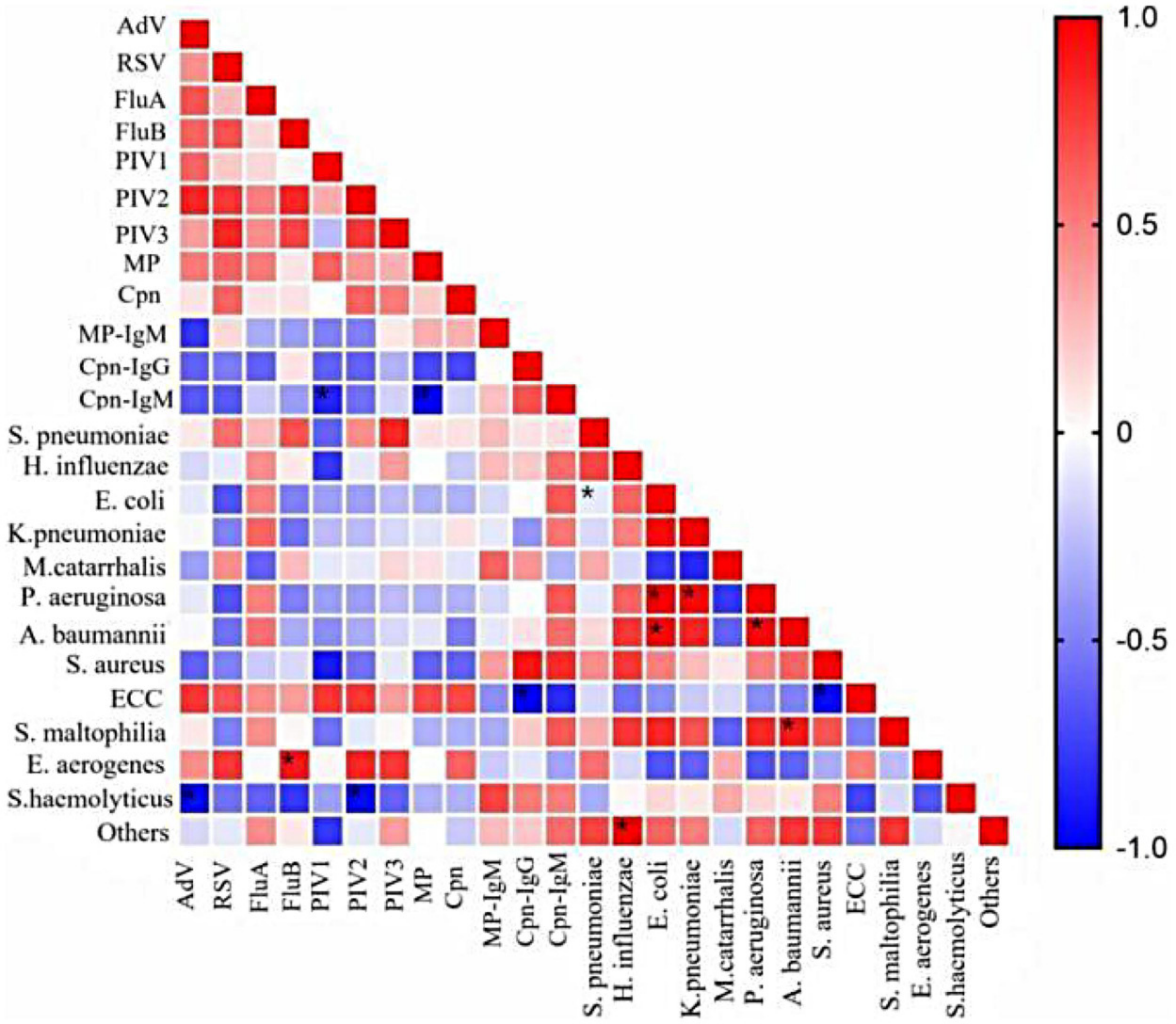
Correlation heatmap of concurrent infections with respiratory pathogens. In this study, Spearman's test was used for correlation analysis. Rows/columns represent different pathogens, and the correlation coefficient between two pathogens ranges from −1 to +1. A larger absolute value and darker color of the square indicate a stronger correlation. Red squares in the figure represent positive correlations, indicating that the co-infection frequency is higher than randomly expected; blue squares represent negative correlations, indicating mutually exclusive infections; white or light-colored squares represent no association, meaning infections occur independently. *Indicates a statistically significant difference (*P* < 0.05). It is worth noting that even if the statistical result is not significant, the effect size of some pathogen combinations (e.g., *Streptococcus pneumoniae*-FluA *φ*=0.25) may still have clinical implications.Co-infection correlation network revealing two distinct ecological patterns: (1) a strong positive correlation cluster among Gram-negative bacteria (e.g., E. coli, K. pneumoniae, P. aeruginosa), suggesting shared risk factors or synergistic biofilm formation; and (2) a mutually exclusive relationship between M. pneumoniae and certain typical bacteria, implying competitive exclusion or divergent clinical presentations.

A correlation analysis was conducted on patients with infections caused by two or more pathogens simultaneously. Due to incomplete data in 2022, this year was not included in the study. In this research, there were six groups of co-infecting pathogens with significant negative correlations, such as Cpn-IgM and PIV1 (*φ*=-0.841, *P* < 0.05), Cpn-IgM and MP (*φ*=-0.975, *P* < 0.05), *S. haemolyticus* and AdV (*φ*=-0.943, *P* < 0.05), and *S. haemolyticus* and PIV2 (*φ*=-0.943, *P* < 0.05). Taking MP, which had the highest number of infections, as an example, MP was more likely to co-infect with viral pathogens but less likely to co-infect with bacterial pathogens. Among them, there was no association between MP and H. influenzae* or E. aerogenes*, while a positive interaction existed between MP and the Enterobacter cloacae* complex(ECC). Significant positive interactions were observed between *E. coli* and K. pneumoniae*, E. coli* and A. baumannii*, K. pneumoniae* and P. aeruginosa*, as well as P. aeruginosa* and A. baumannii* (*φ*>0.9, *P* < 0.05), with details shown in Figure 4.

## 4 Discussion

This six-year follow-up study not only revealed the dynamic changes in pathogens before and after the COVID-19 pandemic but, more importantly, identified the severe infection-related characteristics of different pathogens across various age groups of children, providing an epidemiological basis for early intervention in pediatric critical illness.

Among 51,597 patients, the overall positive rate was 33.90% (17,492/51,597), which was lower than the result reported in other studies in Changsha (11) (97.02%). This discrepancy may be because other studies included patients with recurrent and refractory infections, while this study also included patients without obvious symptoms who sought infection screening, thus better reflecting the epidemiological characteristics of pathogens in the population. Since most of the period from 2020 to 2022 was during the pandemic, “non-pharmaceutical intervention measures” such as population isolation and mask-wearing (12) blocked pathogen transmission to a certain extent, so the positive rate in this study was slightly lower than those reported by other scholars (13). The number of people tested in 2020–2021 decreased due to isolation measures, but the positive rate increased instead. This is thought to be because only people with respiratory infection symptoms sought medical treatment in the early stage of the pandemic, resulting in a higher number of positive patients during this period.

Among common respiratory pathogens, the top three with the highest overall positive rates were MP (49.93%), RSV (25.21%), and PIV3 (8.22%). As the most common atypical pathogen, MP is one of the smallest microorganisms in the world and can spread widely globally with an epidemic cycle of usually 1–2 years (14). After the outbreak of COVID-19 in 2019, the positive rate of MP began to decline, which is similar to the detection results in the southern Anhui region (15). Until 2022, MP (27.35%) lost its dominant position in respiratory infections and was replaced by RSV (31.84%). Notably, in 2023, while the PCR-based detection rate of MP remained low, a significant surge in MP-IgM positivity was observed. This divergence may reflect a shift towards more recent or reinfection cases post-pandemic, where serological responses are prominent.According to national data (6), the common respiratory pathogens covering the pre-pandemic period were influenza viruses (FluA and FluB), RSV, MP, and parainfluenza viruses (HPIV) in sequence. In contrast, the main pathogens in this study before and during the pandemic were MP, RSV, PIV3, and AdV; after the pandemic, they were RSV, MP, PIV3, FluA, and AdV. It can be seen that the composition of pathogen spectra varies across regions, and the COVID-19 pandemic has had a significant impact on changes in pathogen spectra. The main population infected with pathogens in this study was infants aged 0–3 years, which is similar to the research result of Wang (16) (92.8%). Among children under 1 year old, RSV was the main pathogen, accounting for 49.93% (2,202/4,410) of the total patients. Due to the immature immune system in this age group, the positive rates of various pathogens were relatively high; except for MP, the positive rates and number of visits for other respiratory pathogens decreased with the increase of patients' age. MP is more common in older children, and its incidence increases with age, which is consistent with other studies (17, 18).

In this study, most pathogens had a high incidence in autumn and winter and a low incidence in spring and summer, which is consistent with other research results (13, 16). This is also related to the large temperature difference and relatively dry climate in autumn and winter in central and southern China, which easily damage the respiratory mucosa. Among them, AdV and MP had no obvious seasonal specificity and could occur throughout the year, which is consistent with the research results of scholars such as Zhang (19) and Liu (20); both RSV and FluA had the highest incidence in winter and the lowest in summer, which is consistent with other research results in Changsha (11). However, in the study in Shenzhen (16), RSV peaked in summer. This may be because Changsha is closer to the inland and has a cooler climate than Shenzhen, while RSV is more common in winter in temperate climates or rainy seasons in tropical climates (19). However, in 2022 and 2023, RSV and FluA showed an unusual spring-summer prevalence, which we consider to be a delayed rebound after the impact of the pandemic. Although we could not measure serological immunity directly, our age-stratified data provide epidemiological evidence supporting the “immunity debt” hypothesis (21). Notably, children aged 1–3 years in 2023—a cohort born during the strict lockdown period with minimal prior exposure to respiratory viruses—exhibited the most pronounced surge in RSV and PIV3 infections compared to historical controls of the same age group. This cohort-specific vulnerability underscores the consequences of prolonged low-exposure intervals on population susceptibility. Since RSV vaccines have only been on the market in recent years, it is thought to be caused by the RSV immune gap population formed by long-term low exposure (22).

With the introduction of pneumococcal and Haemophilus influenzae conjugate vaccines, respiratory infections caused by bacteria decreased compared with previous years, but they still served as important infection sources and were of great significance for clinical detection (23). To gain deeper insights, this study further investigated patients with bacterial infections and selected 12 clinically common bacteria for statistical analysis: the top three bacteria with the highest detection rates were *S. pneumoniae* (26.35%, 2,104/7,983), *H. influenzae* (20.11%, 1,605/7,983), and *M. catarrhalis* (14.93%, 1,192/7,983). This is different from the research results of scholars such as Li (6) and Wu (24), which is considered to be due to geographical differences and changes in immune function against different bacteria caused by “immune debt” in the body during COVID-19 (21). Further analysis of susceptible bacteria in different age groups showed that the detection rates of *A. baumannii* and *P. aeruginosa* increased in school-age children over 7 years old, and *P. aeruginosa* was more likely to occur in older groups, which was consistent with the research results of Li (6) and other scholars.

To explore whether there is a correlation between infections with different pathogens, a correlation analysis was conducted on relevant data. In this study, it was observed that MP was more likely to co-infect with viral pathogens and less likely to co-infect with bacterial pathogens, which is similar to the research results of other scholars (6). As mentioned earlier, *P. aeruginosa* is more easily detected in older children, and it mostly shows a positive correlation in interactions with other pathogens. Relevant studies have shown that when the body is simultaneously infected with *P. aeruginosa* and *S. aureus*, the quinolone signal (PQS) system can protect *S. aureus* and reduce its sensitivity to vancomycin and other cell wall-active antibiotics (25), so the two bacteria have a co-infection promoting effect in clinical practice; before the pandemic, influenza virus was the dominant virus in most children's respiratory infections, and it also had a co-infection promoting effect with *S. pneumoniae*. Previous scholars have clarified the relevant mechanism: when FluA and *S. pneumoniae* infect the same host, the viral hemagglutinin can regulate bacterial virulence factors, helping bacteria enhance immune evasion (26); due to the lack of methods to simultaneously detect bacteria and viruses, it is difficult to distinguish the specific relationship between bacteria and viruses, and the interrelationships between most pathogens have not been clarified.

This study still has limitations. First, no rare pathogens were involved; second, during the COVID-19 pandemic, the huge workload indirectly promoted the maturity of detection technology, which may have led to an underestimation of the actual positive rate of pathogens in the early stage of the pandemic (27).

## 5 Conclusion

Through a comprehensive analysis of data spanning 2018 to 2023, this study systematically elucidated the long-term impact of the COVID-19 pandemic and the associated non-pharmaceutical interventions on the transmission dynamics and disease spectrum of common respiratory pathogens among children in Central and Southern China. Our findings demonstrated that the pandemic precipitated a marked epidemiological shift, characterized by an initial suppression of pathogen circulation followed by a pronounced resurgence—most notably of RSV, influenza viruses, and Mycoplasma pneumoniae—after the relaxation of containment measures. These temporal patterns lend strong support to the “immunity debt” hypothesis as a key driver of the altered post-pandemic infection landscape.

Importantly, this study delineated age-specific risk profiles that are critical for clinical triage and pediatric intensive care. RSV remained the predominant cause of hospitalization in infants under one year of age, underscoring the urgent need for the integration of newly available RSV prophylactic strategies into routine maternal and infant care. In contrast, school-age children exhibited an increased burden of bacterial co-infections involving opportunistic pathogens such as Pseudomonas aeruginosa and Acinetobacter baumannii, highlighting a vulnerable population that warrants heightened surveillance for severe, complicated pneumonia. Furthermore, the distinct co-infection patterns observed between Mycoplasma pneumoniae and viral vs. bacterial pathogens provide a valuable framework for guiding empirical antimicrobial therapy.

Based on these insights, we propose the following actionable recommendations for future research and public health practice: First, regional surveillance systems should be strengthened to incorporate multiplex pathogen testing for the early detection of unusual seasonal rebounds, thereby enabling timely adjustments to hospital resource allocation and pediatric intensive care unit preparedness. Second, future prospective studies are warranted to elucidate the immunological mechanisms underlying age-dependent susceptibility to specific co-infections, particularly the molecular interplay between respiratory viruses and secondary bacterial invaders. Third, clinical guidelines should emphasize the differential diagnosis of Mycoplasma pneumoniae using both nucleic acid and serological assays during epidemic periods to avoid misclassification of infection status. Collectively, this study not only provides a robust epidemiological baseline for post-pandemic pediatric respiratory care but also offers actionable insights for mitigating the impact of future disruptions to pathogen circulation patterns.

## Data Availability

The raw data supporting the conclusions of this article will be made available by the authors, without undue reservation.
